# Effects of Sub-Chronic Exposure to Imidacloprid on Reproductive Organs of Adult Male Rats: Antioxidant State, DNA Damage, and Levels of Essential Elements

**DOI:** 10.3390/antiox10121965

**Published:** 2021-12-08

**Authors:** Blanka Tariba Lovaković, Vilena Kašuba, Ankica Sekovanić, Tatjana Orct, Antonija Jančec, Alica Pizent

**Affiliations:** 1 Analytical Toxicology and Mineral Metabolism Unit, Institute for Medical Research and Occupational Health, Ksaverska Cesta 2, 10000 Zagreb, Croatia; btariba@imi.hr (B.T.L.); asekovanic@imi.hr (A.S.); torct@imi.hr (T.O.); jancec.antonija@gmail.com (A.J.); 2 Mutagenesis Unit, Institute for Medical Research and Occupational Health, Ksaverska Cesta 2, 10000 Zagreb, Croatia; vkasuba@imi.hr

**Keywords:** antioxidant enzymes, DNA damage, essential elements, glutathione, neonicotinoids

## Abstract

Although considered a good alternative to organophosphate pesticides, there are reports indicating adverse effects of neonicotinoid insecticides on reproduction. Our aim was to assess the effects of exposure to low doses of imidacloprid on antioxidant state, DNA damage, and concentration of essential elements in the testes and epididymis using a rat model. Adult male Wistar rats were orally treated with doses comparable to currently proposed health-based reference values: 0.06 (ADI), 0.80 (10× AOEL), or 2.25 (1/200 LD_50_) mg/kg b.w./day for 28 consecutive days. Exposure to 2.25 mg/kg b.w./day of imidacloprid resulted in a significantly lower testis weight (1.30 ± 0.17 g compared to 1.63 ± 0.15 g in controls). Treatment with 0.06 mg/kg b.w./day increased the level of reduced glutathione in the epididymis (73%), while the activities of epididymal glutathione peroxidase and superoxide dismutase significantly increased in all treated rats (74–92% and 26–39%, respectively). Exposure to imidacloprid resulted in a low, but significant, level of DNA damage in testicular sperm cells regardless of the concentration applied (<28% compared to the negative control). Higher concentrations of Mo were measured in the testes of rats treated with 0.80 and 2.25 mg/kg b.w./day (72.9 ± 7.9 and 73.9 ± 9.1 mg/g, respectively) compared to the control animals (60.5 ± 7.8 mg/g). Higher concentrations of Na were measured in the testes of rats treated with 2.25 mg/kg b.w./day (1679 ± 82 mg/g compared to 1562 ± 56 mg/g in controls). The fact that such low doses of imidacloprid were able to produce measurable biological effects calls for the further evaluation of this widely used insecticide.

## 1. Introduction

Neonicotinoid pesticides are nowadays one of the most important systemic insecticide groups used worldwide, accounting for more than 25% of the global pesticide market [1]. They act as neurotoxins by blocking nicotinic acetylcholine receptors (nAChR) and preventing acetylcholine from transmitting impulses between nerves, resulting in the insect’s paralysis and eventual death. The first neonicotinoid registered for use as a pesticide by the United States Environmental Protection Agency (US EPA) was imidacloprid ([1-(6-chloro-3-pyridylmethyl)-N-nitroimidazolidin-2-ylideneamine]), mainly applied in agriculture to prevent and control piercing and sucking insects, as well as in many veterinary drugs for flea control in pet animals [2]. In 2016, pest management professionals used approximately 136,000 kg of imidacloprid, while around 18,000 kg of imidacloprid was purchased directly by consumers for indoor and outdoor use [3]. 

Imidacloprid has a relatively long half-life in soil and high-water solubility, which contributes to its persistence and transport in the environment. It has been frequently detected in soils and sediments, but also in water and food products (reviewed in [2]), indicating several possible routes of human exposure. Once viewed as an ideal replacement for organophosphate and carbamate pesticides, the pervasiveness of imidacloprid in the environment has raised global concern regarding toxic effects on non-target species. Imidacloprid has a selective affinity for insect nAChRs over those belonging to vertebrates [4], and its acute toxicity is considered relatively low to mammals. However, there is increasing evidence that long-term exposure could cause toxic effects in non-target species. These include neurotoxic, immunotoxicity, and hepatotoxic effects, as well as impaired reproductive function [5,6,7,8,9]. Although only a few animal studies have tried to evaluate the risks of exposure for male reproduction, findings clearly demonstrate the potential of imidacloprid to cause reproductive toxicity (reviewed in [10]). Sub-chronic and chronic exposure to imidacloprid can result in histological damage of the testicular tissue, disruption of sex hormones, and impairment of spermatogenesis accompanied by changes in sperm morphology, increased sperm mortality, and decreased sperm count and motility, all of which could affect fertility [6,11,12,13,14].

The generation of reactive oxygen species (ROS) has been described as the most important event that leads to adverse reproductive health outcomes of exposure to imidacloprid [6,7,8,9,10]. The ROS are constantly produced during normal cellular energy production and metabolism in the mitochondria. Physiological concentrations of hydroxyl radical (•OH), superoxide anion (O_2_^•−^), and hydrogen peroxide (H_2_O_2_) have important roles in cellular regulation through signal transduction pathways and gene expression involved in cell metabolism, growth, development, and differentiation. However, elevated levels of these species contribute to the detrimental changes during spermatogenesis, epididymal maturation, and sperm capacitation leading to infertility [15,16]. Pesticide-induced oxidative stress in the testes and epididymis is a possible mechanism of its reproductive toxicity manifested through multi-step pathways that are still only partly understood [17]. The reproductive system is equipped with antioxidant enzymes and non-enzymatic free radical scavengers, which act interactively in the prevention of induction and/or reduction of oxidative stress [16,18,19]. The primary antioxidant enzymes involved in ROS scavenging are superoxide dismutase (SOD), glutathione peroxidase (GPx), and catalase (CAT). SOD is highly important in this defense and is present in cytosolic, mitochondrial, and extracellular forms in the testis. SOD eliminates the superoxide radical (O_2_^•−^) by converting it to hydrogen peroxide, which is then converted in water by CAT and GPx. GPx is considered to be a key enzyme in the regulation and prevention of oxidative damage that can be caused by hydrogen peroxide. Together with non-enzymatic scavengers such as glutathione (GSH), enzymes work to maintain the ROS at physiological levels and avoid the adverse effects of oxidative stress [20]. GSH is able to eliminate free radicals or deal directly with the causes of oxidative stress such as pesticides. GSH participates in the reduction of hydrogen peroxide in the reactions catalyzed by GPx, readily reacting with hydroxide ion [21]. Oxidative stress induced by imidacloprid could disrupt antioxidant balance and promote detrimental effects in testes and epididymis and disturb sperm maturation and DNA compaction. The spermatozoon membrane contains high amounts of polyunsaturated fatty acid, while its cytoplasm has inadequate antioxidant capacity, which makes spermatozoa highly vulnerable to ROS and lipid peroxidation [22,23].

The majority of studies of imidacloprid toxicity in rats were conducted at doses higher than 16 mg/kg b. w. [12,24,25]. The exposure of rats to higher doses of imidacloprid (>16 mg/kg b. w.) resulted in elevated lipid peroxidation and lower levels of glutathione (GSH) and antioxidant enzymes in testicular tissue, confirming oxidative stress as the mechanism of imidacloprid’s reproductive toxicity [12,24]. The only studies of sub-chronic low-level exposure, and the first to suggest the mechanisms of toxic effects on male reproduction, are studies by Bal et al. [6,7], where the exposure of developing and adult rats to imidacloprid at doses lower than 8 mg/kg was followed by the induction of oxidative stress in the testes and the impairment of testicular function. The threat of oxidative damage is particularly significant to DNA. Several in vitro and in vivo investigations of imidacloprid genotoxicity confirmed a dose dependent relationship between the concentration of imidacloprid and DNA damage [26,27,28,29,30]. ROS may cause damage to base exchange (DNA-protein crosslinking and DNA chain rupture) while the generated superoxide free radicals and alkyl radicals may further intensify DNA damage or even change gene expression [31].

The widespread use of imidacloprid leads to exposure of vulnerable populations, including men and women of reproductive age. Therefore, it is important to investigate its impact on reproductive health. This study aimed to assess the potential effects of exposure on the reproductive organ system of adult male Wistar rats at levels relevant for real-life scenarios set out in the current EU legislation and in general considered not harmful to humans. Given the possible toxic effect of imidacloprid on male reproduction, we determined whether and how a repeated 28-day oral exposure affected antioxidant defense and primary DNA damage in testicular and epididymal tissue. The levels of glutathione (GSH) and activities of antioxidant enzymes, GPx and SOD, were measured in the adult male testes and epididymis as indicators of oxidative stress response. In addition, we measured the tissue concentration of essential elements, as many of them are cofactors of enzymes and metalloproteins involved in a wide range of biological processes, including spermatogenesis, DNA metabolism and repair, and gene expression [32]. DNA strand breaks were analyzed by the modified comet assay, i.e., alkaline/neutral as endpoints in adult male rat testicular/epididymal cells to evaluate primary DNA damage.

## 2. Materials and Methods

### 2.1. Tested Compound

The tested substance Imidacloprid (CAS number 138261-41-3) was purchased at an analytical standard from Pestanal^®^ quality (a registered trademark of Sigma-Aldrich Laborchemikalien GmbH, Seelze, Germany). All other chemicals and reagents used for biochemical analysis and in the alkaline comet assay were of analytical grade and purchased from Sigma Chemical Co., St. Louis, MO, USA, unless specified otherwise. 

### 2.2. Animals and Animal Husbandry 

The experiment was conducted on three-month old male Wistar HsdBrlHan rats (*Rattus norvegicus* sp.) (mean ± st.dev.: 250 ± 11 g b. w.; median and range: 248 (230–274) g b. w.) obtained from the Animal Breeding Unit of the Institute for Medical Research and Occupational Health (IMROH), Zagreb (Croatia). Animals were kept in clear polycarbonate cages with 40–60% humidity at 22 °C and normal 12-h light/dark cycle. All of the animals had free access to standard pellet feed for small rodents (4RF 21, Mucedola, Settimo Milanese, Italy) and tap water. At the start of the study, rats were weighed and inspected by a licensed veterinarian at IMROH. The Institutional Animal Care and Use Committee and the Croatian Ministry of Agriculture approved the study (Reg. no. 100-21/14-5, Class 01-18/14-02-2/6 of 11 June 2014). The experiment was conducted in line with the EU Directive 2010/63/EU for animal experiments and in compliance with international standards and national legislation to protect animal welfare.

### 2.3. Experimental Design 

The selection of imidacloprid doses was based on the reference values according to the EFSA [33], WHO [34], and EU Pesticides Database [35] to include environmentally relevant levels, usually not harmful to humans. A total of 30 rats were randomly assigned to control and treatment groups. Each group comprised five animals with minimal weight variation. Imidacloprid was orally administered for 28 consecutive days to three groups of animals at doses of 0.06 mg/kg b. w./day (Acceptable Daily Intake—ADI), 0.8 mg/kg b. w./day (10-fold higher than Acceptable Operator Exposure Level—AOEL), and 2.25 mg/kg b. w./day (equal to 1/200 acute oral LD_50_ for rats). Imidacloprid solutions for treatments were prepared freshly before use. The stock solution was prepared by dissolving the test compound in 0.03% ethanol (EtOH), and final solutions for application were dissolved in distilled water.

Negative control animals received tap water, and solvent control animals received 0.03% EtOH solution for 28 days. All rats treated with imidacloprid received the same exposure to EtOH (0.03%). Appropriate positive controls received ethyl methanesulphonate (EMS), a well-established genotoxicant recommended for in vivo comet assay in rodents [36], at 300 mg/kg b. w./day *p.o.* the last three days of the experiment. All groups of animals were handled in the same manner.

During the experiment, body weights were regularly monitored on a weekly basis, and the volume/concentration of applied imidacloprid solution was adjusted accordingly. Survival and clinical signs of intoxication were evaluated on a daily basis.

### 2.4. Collection of Testes and Epididymis

All of the animals were humanely euthanized 24 h after the last treatment by exsanguination under Xylapan/Narketan anaesthesia (Narketan, Vetoquinol UK Ltd., Towcester, UK, 80 mg/kg b. w.; Xylapan, Vetoquinol UK Ltd., 12 mg/kg b. w., *i.p.*). A licensed veterinarian at IMROH examined gross pathological changes of the internal organs in all of the animals. 

Testes and epididymis were dissected, cleaned from adhering matters, and rinsed in cold PBS buffer (without Ca^2+^ and Mg^2+^). The tissues were symmetrically bisected and weighed. The left testis/epididymis was stored in cryo-tubes without additional media or cryoprotectants, while the right testis/epididymis was symmetrically halved and stored separately. One-half was used for the measurements of antioxidant status parameters and the other one for element analysis. The tubes were immediately frozen in liquid nitrogen and stored at −80 °C until use. 

### 2.5. Determination of the Antioxidant Status 

Before the measurement of antioxidant status parameters, the tissue samples were homogenized (100 mg tissue/mL 50 mM phosphate buffer, pH 7.8) and centrifuged at 20,000× *g* and 4 °C for 30 min to obtain a supernatant. 

Glutathione peroxidase (GPx) activity in tissue supernatants was determined spectrophotometrically according to the method described by Belsten and Wright [37]. Briefly, 50 µL of supernatant was diluted with 500 µL of DL-Dithiothreitol (0.1 mol/L). After 5 min stabilization, samples were further diluted (10 times) with double strength Drabkin’s reagent and kept at 4 °C until analysis. Portions of 0.8 mL of reaction mixture (containing 0.1 mmol phosphate buffer pH 7.0, 0.01 mmol EDTA-Na_2_ (Merck, Darmstadt, Germany), 1 EU glutathione reductase, 5 µmol of GSH, and 0.25 µmol β-NADPH) and 100 µL of diluted sample were pipetted into a measurement tube, and the reaction was initiated with 0.1 mL *t*-butyl hydroperoxide (2.5 µmol). The amount of GSH oxidized by *t*-butyl hydroperoxide was determined by following the decrease in the β-NADPH concentration, and the decrease in absorbance was measured at 340 nm (Cary 50 UV-Vis, Varian Inc., Palo Alto, CA, USA). One unit of GPx is the number of micromoles of β -NADPH oxidized per minute. The results were expressed as IU/g protein. Protein content was measured by the Bradford assay (Bradford, 1976), using bovine serum albumin as the standard.

Superoxide dismutase (SOD) activity in tissue supernatants was measured by the European standardized method [38] using the Ransod kit (Randox, Crumlin, UK) according to the manufacturer’s instructions. This method employs xanthine and xanthine oxidase to generate superoxide radicals that react with 2-(4-iodophenyl)-3-(4-nitrophenol)-5 phenyltetrazolium chloride (I.N.T.) to form a red formazan dye. SOD activity was then measured by the degree of inhibition of this reaction at 505 nm by spectrophotometry (Cary 50 UV–Vis, Varian Inc., Palo Alto, CA, USA). One unit of SOD is that which causes a 50% inhibition of the rate of reduction of I.N.T. under the conditions of the assay. SOD activity was expressed as IU/g protein. 

Glutathione (GSH) was analyzed in tissue supernatants using the method by Ellman [39]. Proteins were removed by adding 100 μL of 5% trichloroacetic acid to 300 μL of supernatant. The homogenates were then mixed and centrifuged for 10 min at 1300× *g*. 850 μL of phosphate buffer and 50 μL of DTNB were added to 100 μL of H_2_O (blank solution), standards, or supernatant of homogenates. The absorbance of blank solution, standards, and samples were measured spectrophotometrically (Cary 50 UV-Vis, Varian Inc., Palo Alto, CA, USA) at 412 nm. GSH concentration was calculated from the calibration curve of standards and expressed as micrograms per gram of tissue.

### 2.6. Multi-Element Analysis

For elements analysis tissue samples (~1 g for testis, 0.2–0.4 g for epididymis) were digested with nitric acid and ultrapure water (1:1) using the UltraCLAVE IV (Milestone, Sorisole, Italy) microwave digestion system. After digestion, testis samples were adjusted to 5 g and epididymis to 4 g with ultrapure water (GenPure, TKA System GmbH, Niederelbert, Germany) and stored at room temperature until analysis.

Element concentrations (Na, Mg, Ca, K, Fe, Cu, Zn, Se, Mo, and Mn) in the testis and epididymis were determined by an inductively coupled plasma–mass spectrometer (ICP-MS) on Agilent 7500cx (Agilent Technologies, Tokyo, Japan) equipment with I-AS autosampler, collision/reaction cell, Ni sampler, and skimmer cones (orifice diameter of <1.0 and <0.4 mm), MicroMist nebulizer, spray chamber (Scott–type) and torch (2.5 mm diameter injector) with the Shield Torch system. The cell was pressurized with a gas, such as helium or hydrogen (UTP d.o.o., Zagreb—SOL Group, Italy; purity of >99.9999%); helium mode was used for ^23^Na, ^24^Mg, ^39^K, ^43^Ca, ^55^Mn, ^56^Fe, ^63^Cu, ^68^Zn, and ^95^Mo, while ^78^Se was measured in hydrogen mode. The tuning solution of 1 µg/L of ^7^Li, ^59^Co, ^89^Y, ^140^Ce, ^205^Tl and ^78^Se was used to optimize the instrument to obtain the highest signal-to-background ratio. Prior to element analysis, samples were diluted to 1:6 with 1% (*v*/*v*) HNO_3_ and 3 µg/L internal standards (^74^Ge, ^103^Rh, ^175^Lu and ^193^Ir). Calibration standards and reference materials were prepared in the same manner as the samples. The accuracy of measurements was checked by reference materials (bovine liver NIST 1577, 1577a and 1577b, bovine liver BCR 185R, and pig kidney BCR 186R), and recovery ranged from 93 to 116%. Preparation and analysis of samples were performed in a laboratory with an HVAC system (Heating, Ventilating, Air Condition) and HEPA filters.

### 2.7. Evaluation of DNA Damage

Instead of the standard alkaline comet assay, an “alkaline/neutral protocol” (i.e., alkaline DNA unwinding followed by electrophoresis in neutral condition) [40,41] for the testes and epididymis was used according to the method described in Tateno and Kamiguchi [42].

The frozen tissue in cryo-tubes was first quickly thawed in a water-bath at 37 °C and ice-cold PBS (free of Ca^2+^ and Mg^2+^) was added. Samples were minced with fine scissors, left for a few minutes for coarse particles to settle to the bottom, and the supernatant was collected with a Pasteur pipette. 

Clear microscope slides (Vitrognost Ultra Plus) were pre-coated with 1% normal melting point agarose in water and dried at 50 °C on a heating plate. In total, 300 µL of 1% low melting point (LMP) agarose in PBS was added on pre-coated slides. The prepared cells in PBS were mixed with the 1% LMP agarose to a final concentration of 0.7%. This suspension was dropped on top of the first layer and covered with a cover slide. Slides were placed on a metal tray over ice (at 4 °C) for 10 min so the gel could solidify. Cover slides were then removed, and slides were submerged in cold lysis buffer (2.5 M NaCl, 50 mM Na_2_-EDTA, 10 mM Tris, 10% DMSO (DMSO is added to a lysis solution as a protectant against free radicals within the lysis solution), 1% Triton X-100, pH = 10) at 4 °C for 2 h, protected from light. Afterwards, the slides were incubated in fresh lysis solution (2.5 M NaCl, 50 mM Na_2_-EDTA, 10 mM Tris, 1% Triton X-100, and 10 mM dithiothreitol, pH = 10) at 37 °C for 1 h, protected from light. Following the second cell lysis, ice-cold re-distilled water was used to wash each slide three times at three-minute intervals in order to remove salt and detergent from microgels. The slides were then immersed for one minute in ice-cold 300 mM NaOH supplemented with 1 mM Na_2-_EDTA and transferred to Tris acetate-EDTA buffer (TAE) for neutralization (10 min). Then, the slides were subjected to electrophoresis in horizontal electrophoresis tank (Horizon 11.14, Analytikjena, Biometra GmbH, Gottingen, Germany) for 10 min (0.5 V/cm, 10 mA) at room temperature in TAE buffer. 

After electrophoresis, slides were washed in distilled water and dehydrated in 70 and 96% EtOH (10 min in each), dried, and stored in plastic box (protected from moisture and light). The air-dried slides were stained with ethidium-bromide (20 µg/mL). From each animal, two replicate slides were prepared with 150 comets per slide. Slides were analyzed by a fluorescent microscope (Olympus BX51) connected to a CCD camera and software Comet Assay IV (Perceptive Instruments Ltd., Suffolk, Halstead, UK). As a descriptor for the DNA damage, tail intensity (DNA% in the tail of the comet) was used. Fragments of damaged DNA move at different speed in the electrical field and create a ”comet” pattern because they have different molecular weights and different electrical charges due to damage [43]. The edges of the gel and superimposed comets were avoided. During the scoring of testes cell gels, two sizes of nucleoids were observed, and they were scored separately for each slide. The smaller nucleoid was presumed to have one half of DNA (haploid cell), and the bigger nucleoid was presumed to be a diploid cell [44].

### 2.8. Statistical Analysis

Statistical analysis was run on a Dell™ Statistica™ licensed statistical software package Version 13.5.0.17 (TIBCO Software Inc., Palo Alto, CA, USA). Statistical significance was set at *p* < 0.05. Each group comprised five animals, and the described experiment was conducted once. 

Normality of distribution was tested with the Kolmogorov–Smirnov test. The data obtained for organ (testis and epididymis) weight and oxidative stress parameters were normally distributed, while other data (concentration of elements and DNA damage) were not. Organ weights were analyzed with One-way ANOVA, followed by *post-hoc* Tukey’s HSD test. The results were expressed as means ± standard deviation. As the results of element concentrations and the alkaline comet assay were not normally distributed, we used the Kruskal–Wallis test followed by a multiple comparison of mean rank for all groups. The results were expressed as median and range (min-max).

## 3. Results

After 28 days of treatment, there were no deaths or signs of systemic toxicity among the animals. The applied doses of imidacloprid did not produce marked behavioral changes between exposed and control groups, while gross necropsy did not reveal any treatment-related findings.

### 3.1. Changes in Weight of Testes and Epididymis

**Figure 1:**
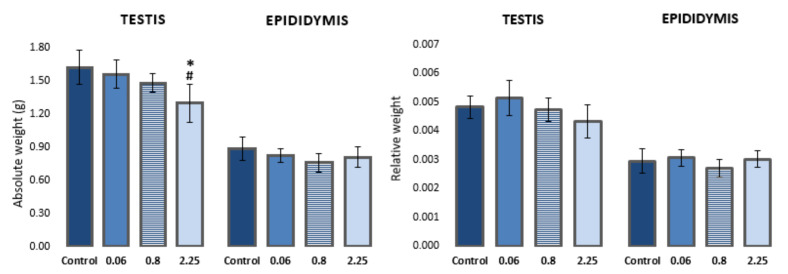
Absolute and relative weights of the left testis and epididymis of Wistar rats (N = 5 rats per group) treated orally for 28 consecutive days with imidacloprid at doses of 0.06 mg/kg b. w./day, 0.8 mg/kg b. w./day, and 2.25 mg/kg b. w./day and the respective negative control. Results are presented as means ± standard deviation. Statistical significance was set at *p* < 0.05. *—vs. negative control, #—vs. dose 0.06 mg/kg b. w./day.

A dose-dependent effect on body weight gain was observed in rats during treatment with imidacloprid. Animals treated with higher doses of imidacloprid had lower body weight gain in comparison to control animals (reported in [30]). Animals treated with the highest dose of imidacloprid (2.25 mg/kg b. w./day) had a significantly lower absolute weight of testes compared to negative controls and animals treated with 0.06 mg/kg b. w./day (F = 5.282, *p* < 0.05; One-way ANOVA). However, no significant difference was observed in the relative weight of testes (testis-to-body weight ratio) between the groups. There were no statistically significant differences in the absolute/relative weight of epididymis between the groups (Figure 1, Table S1).

### 3.2. Antioxidant Status

**Figure 2:**
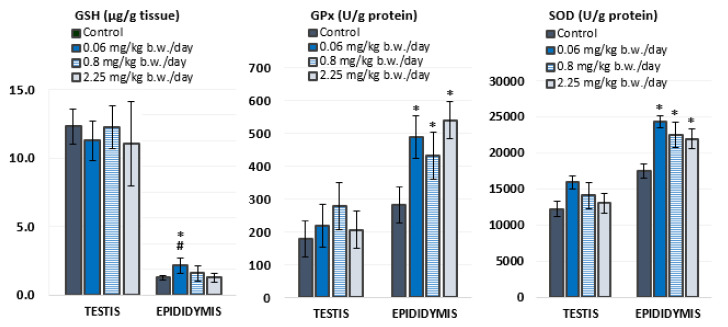
Parameters of antioxidant status measured in the testicular and epididymal tissue of Wistar rats (N = 5 rats per group) treated orally for 28 consecutive days with imidacloprid at doses of 0.06 mg/kg b. w./day, 0.8 mg/kg b. w./day, and 2.25 mg/kg b. w./day and in the negative control group. GSH—levels of glutathione; GPx—glutathione peroxidase activity; SOD—superoxide dismutase activity. Results are presented as means ± standard deviation. Statistical significance was set at *p* < 0.05 (*—vs. negative control, #—vs. dose 2.25 mg/kg b. w./day).

Antioxidant status was evaluated in the testis and epididymis of experimental animals by measuring levels of GSH and activities of antioxidant enzymes, GPx and SOD (Figure 2, Table S1). In general, the testes contained higher levels of GSH than the epididymis, while the activity of both antioxidant enzymes was higher in the epididymis than in the testes. 

Oral exposure to imidacloprid during 28 consecutive days did not cause significant changes in oxidative stress response in the testes. However, rats treated with 0.06 mg/kg of imidacloprid b. w./day had significantly higher levels of GSH in the epididymis compared to negative controls and rats treated with 2.25 mg/kg b. w./day of imidacloprid. Significantly higher activities of GPx (F = 12.035, *p* < 0.001; One-way ANOVA) and SOD (F = 10.781, *p* < 0.001; One-way ANOVA) were measured in the epididymis of all groups treated with imidacloprid in comparison to negative controls. These results did not display a linear dose–response relationship.

### 3.3. Concentration of Essential Elements in Testis and Epididymis

The concentration of ten essential elements (Na, Mg, Ca, K, Fe, Cu, Zn, Se, Mo, and Mn) measured in the testes and epididymis of control and imidacloprid-treated rats is reported in Table 1 and Table S1. A significantly higher concentration of Na was measured in the testis of rats after 28-day oral exposure to 2.25 mg/kg b. w./day of imidacloprid in comparison to negative controls and rats exposed to 0.06 mg/kg b. w./day. Rats exposed to 0.8 or 2.25 mg/kg b. w./day of imidacloprid had a significantly higher concentration of Mo in the testes than negative controls and rats exposed to 0.06 mg/kg b. w./day of imidacloprid. Treatment with imidacloprid had no effect on the concentration of essential elements in the epididymis of rats.

### 3.4. The Alkaline Comet Assay

**Figure 3:**
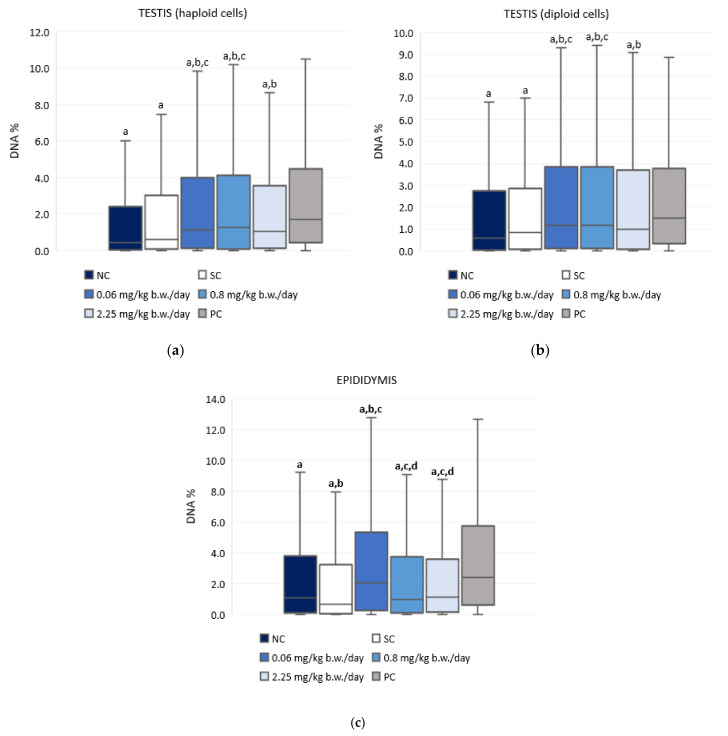
Results of the alkaline comet assay expressed as tail intensity (DNA%) in (**a**) testicular haploid, (**b**) testicular diploid, and (**c**) epididymal sperm cells of Wistar rats (N = 5 rats per group) treated orally for 28 consecutive days with imidacloprid at doses of 0.06 mg/kg b. w./day, 0.8 mg/kg b. w./day, and 2.25 mg/kg b. w./day and in the respective controls (NC—negative control, SC—solvent control, PC—positive control). Data are presented as median (line), 25th and 75th percentile (box), and range (whisker). Statistical significance was set at *p* < 0.05 (a—vs. positive control, b—vs. negative control, c—vs. solvent control, and d—vs. dose 0.06 mg/kg b. w./day).

Figure 3 and Table S1 present the results of the alkaline comet assay expressed as comet tail intensity (DNA%) measured in the haploid and diploid testicular sperm cells and the epididymal sperm cells of male Wistar rats after a 28-day oral exposure to imidacloprid. Although treatment with imidacloprid caused a low level of DNA damage in testicular cells, the observed differences between imidacloprid-treated groups and the negative control were statistically significant. However, there was no dose-related increase of DNA damage. Treatment with 0.06 mg/kg b. w./day of imidacloprid resulted in significantly higher DNA damage observed in epididymal sperm cells compared to the negative and solvent control group. Tail intensity measured in the epididymal sperm cells of rats treated with 0.06 mg/kg b. w./day of imidacloprid was also significantly higher than in rats treated with doses of 0.8 or 2.25 mg/kg b. w./day. The tail intensities measured in testicular and epididymal sperm cells of all imidacloprid-treated rats were lower compared to the positive control. 

## 4. Discussion

Despite increased concern regarding the eco-toxicological effects of imidacloprid, there is a lack of information on the negative effects that imidacloprid may render on mammalian reproductive systems. Experimental conditions in the majority of the existing animal studies included relatively high concentrations of imidacloprid (>16 mg/kg b. w.), so we aimed to investigate the effects that may occur after sub-chronic oral exposure at the concentration levels of imidacloprid potentially encountered in everyday life. These concentrations were calculated based on several toxicological reference values (acceptable daily intake, acceptable operator exposure level, and acute oral LD_50_) and were applied orally in adult rats, considering that the diet is the main route of human exposure. We analyzed testicular and epididymal tissue biomarkers as the endpoints that can be disrupted after imidacloprid ingestion. The main finding of the present study was that imidacloprid applied at low concentrations had the potency to inflict primary DNA damage to both testicular and epididymal cells and trigger oxidative stress response in epididymal cells, although in a non-linear manner. 

### 4.1. Effect on Weight of Testis and Epididymis

A decrease in rat weight gain was observed, which was statistically significant at the highest applied dose (2.25 mg/kg b. w./day) compared to the weight gain of all other experimental groups (reported in our recent paper, [30]). The weight of the epididymis did not differ significantly between groups, but in accordance with the results for the animal weight gain, the absolute weight of the testis was significantly lower in rats treated with the highest dose of imidacloprid compared to the negative control group and rats treated with the lowest dose of imidacloprid. Sub-chronic exposure of adult male Wistar rats to the ten-times higher dose (22.5 mg/kg b. w./day) also resulted in a significant decrease in weight gain, decrease in the relative and absolute weight of the testis, and relative weight of the epididymis compared to the controls [14,25]. The authors stated that the lower testicular and epididymal weight is a direct consequence of the decrease in plasma testosterone and total protein content, considering that these organs require a continuous androgenic stimulation for their normal growth and functions [6]. It was previously observed that nicotine and its agonists could inhibit 17-α hydroxylase, thus preventing the conversion of pregnenolone and progesterone into appropriate precursors and inhibiting testosterone synthesis in testicular cells [45]. Such an effect of imidacloprid on the reduction of testosterone has been confirmed in several animal studies [6,7,11,12,14].

### 4.2. Oxidative Stress Response

In addition to the disruption of steroidogenesis, exposure to imidacloprid may lead to the generation of ROS and RNS and the induction of oxidative stress. The enzymatic antioxidant defense system plays a critical role in protecting cells from reactive species and includes enzymes SOD, catalase (CAT), GPx, glutathione reductase (GR), and glutathione S-transferase (GST). These enzymes serve as good redox biomarkers, as they are the first-line indicators of the antioxidant state through oxidation/reduction processes together with GSH, which acts as a substrate in enzymatic reactions involving GPx, GR, and GST [46]. 

Although exposure to imidacloprid significantly altered biochemical variables indicative for oxidative stress in the epididymis at all tested doses, there was no significant response in the testis. The increase in the activity of antioxidant enzymes indicates that exposure to imidacloprid caused moderate toxicity in the epididymis, while the absence of the effect of imidacloprid on the measured parameters in the testis can be attributed to the very low doses applied in this study. A recent study by Tetsatsi et al. [25] reported a significant increase in the activity of antioxidant enzymes SOD, CAT, and total peroxidases in the testes of adult male rats after 14 days of treatment with a commercial formulation of imidacloprid (Colibri^®^) at a dose of 22.5 mg/kg b. w./day. However, results of several other studies reported a decrease in the concentration of GSH and the activity of antioxidant enzymes (CAT, SOD, GPx and GST) in the testes of rats exposed to different doses of imidacloprid [6,7,12,14,24]. In these studies, imidacloprid treatment resulted in a significant reduction in enzyme activity in the testis, possibly due to enzyme inactivation upon exposure to higher concentrations of imidacloprid and excessive ROS formation in the tissue [12,14,24] or longer period of exposure [6,7]. 

In this study, the epididymis was shown to be more sensitive to oxidative changes caused by exposure to low doses of imidacloprid than the testes. Similar findings were obtained recently by Wu et al. [47]. The authors reported that after 9 weeks treatment of adult male rats with *tert*-butyl hydroperoxide, the epididymis, in contrast to the testis, was affected by the treatment, displaying markers of oxidative stress. In mammals, the epididymis has an important role in sperm maturation and storage. The specific microenvironment of the epididymis protects gametes until ejaculation and affects the regulation of epididymal function and integrity. Due to the high content of polyunsaturated fatty acids and the scarce amount of cytoplasm, sperm cells are very sensitive to oxidative damage, which can result in impaired sperm function [48]. The epididymis is well equipped with an antioxidant system that protects spermatozoa against oxidative damage [23], and the GPx enzyme group has the most important function in protecting and preserving their fertilizing capacity during maturation and storage in the epididymis [49]. In addition, the mRNA of the CuZnSOD enzyme is expressed at very high levels along the entire epididymis and does not differ significantly between different regions of this organ [50]. However, compared to the highly efficient blood-testis barrier, the tight junctions of the epididymis appear to be much less exclusive and therefore effective [51]. The observed increase in GSH concentration levels and the catalytic activity of GPx and CuZnSOD in the epididymis was most likely the result of an adaptive response to low levels of oxidative stress. Low concentrations of the toxicant stimulate the synthesis of the repair system and the activation of antioxidant defense mechanisms. Therefore, exposure to imidacloprid and, consequently, the generation of ROS may increase the rate of GSH synthesis and the content of GSH [52].

### 4.3. Effect on Levels of Essential Elements

Trace elements are of the utmost importance for the maintenance of a healthy status of an entire organism. There is a close relationship between levels of certain elements and human reproductive function. Trace elements such as Ca, Na, K, Mg, Cu, Zn, and Mn are necessary for normal spermatogenesis, sperm maturation, motility and capacitation, and normal sperm function [53]. Among them, cations Na, K, and Ca are involved in the regulation of ion balance in spermatozoa, which has been shown to be essential for sperm motility and fertility [54]. In the current study, a significantly higher concentration of Na was measured in the testes of rats after 28-day oral exposure to the highest dose of imidacloprid compared to the controls. Although not statistically significant, a similar increase can also be perceived in the epididymis. A possible explanation is that the exposure to imidacloprid resulted in a disturbance in ion-transport mechanisms at the cell plasma membrane. Active Na^+^ and K^+^ exchange is under the control of the Na, K-ATPase, which is responsible for maintaining the low intracellular Na^+^ concentration [55]. In mammalian cells, there are four different isoforms of the Na, K-ATPase catalytic subunit (NKAα1, NKAα2, NKAα3, and NKAα4) with NKAα4 being the isoform produced solely in male germ cells of the testis [54]. A study by Jimenez et al. [55] reported that sperm lacking NKAα4 manifests ion balance changes, high intracellular Na^+^ levels, and membrane depolarization, which may seriously affect sperm motility and fertility.

On the other hand, a significantly increased concentration of Mo measured in the testicular tissue of rats exposed to doses of 0.8 and 2.25 mg/kg b. w./day of imidacloprid were most likely related to the oxidative stress response. Molybdenum is an essential trace element in animals and humans, where it has been identified as part of the active sites of several enzymes such as xanthine dehydrogenase and aldehyde oxidase. All enzymes that depend on Mo catalyse redox reactions with the help of its versatile redox chemistry, which is controlled by the cofactor itself and the enzyme environment [56]. Zhang et al. [57] have previously reported beneficial effects of low Mo concentrations on the reproductive system of adult female mice. However, the mechanisms by which Mo improves the antioxidant defense remains to be elucidated. Epidemiological studies have also indicated that exposure to Mo is associated with reduced levels of serum testosterone [58,59].

### 4.4. Genotoxic Effects of Exposure to Imidacloprid

Existing in vivo studies on the potential genotoxicity of imidacloprid report increased primary DNA damage in coelomocytes of the earthworm *Eisenia fetida* [60] and in the liver from tree frogs [61] and rats [62]. Ahmed and Nasr [63] treated rats for 28 days with 80 mg/kg imidacloprid and found increased levels of 8-hydroxydeoxyguanosine, indicating DNA fragmentation. We recently demonstrated that oral 28-day exposure of rats to low doses of imidacloprid directly induced DNA damage in leukocytes and brain cells with slight changes in plasma oxidative stress parameters [30]. Although imidacloprid is not classified as genotoxic to humans, there are several in vitro studies of its genotoxic activity in human cells (neuroblastoma (SH-SY5Y) and hepatocellular carcinoma (HepG2) cells and peripheral lymphocytes) reporting genotoxic effects at μM-concentrations [26,27,28,29,64,65]. Calderon-Segura et al. [27] observed significant dose-dependent cytotoxic and genotoxic effects in human peripheral lymphocytes exposed for 2 h to imidacloprid over a wide dose range. The authors suggested that neonicotinoid insecticides, such as imidacloprid, are direct genotoxic agents that could act as a source of free radicals or ROS in exposed human cells and produce DNA damage in the form of single- and double-strand DNA breaks and nucleoside modifications. Similarly, Feng et al. [29] reported significantly increased micronuclei and sister chromatid exchange frequencies and DNA damage in human lymphocytes treated with imidacloprid at concentrations ranging from 0.05 µg/mL to 0.5 µg/mL following a 1 h treatment. Senyildiz et al. [64] observed significant changes consistent with DNA damage at 500 μM in SHSY-5Y cells, as well as alterations in HepG2 cells after 24 h exposure. On the other hand, Costa et al. [28] proposed that concentrations below 20 μM were not genotoxic to human lymphocytes in vitro. Želježić et al. [65] also reported that low concentration exposure (0.13–0.314 µg/mL) within 24 h did not induce primary DNA damage in human lymphocytes.

However, there is a lack of information on what concerns imidacloprid genotoxic effects on male gametes. With the exception of an animal study by Bal et al. [6], there is no similar study. Bal et al. [6] observed apoptosis and fragmentation of rat seminal DNA after treatment with 2 and 8 mg/kg of imidacloprid for 90 days. Oxidative stress, deficiencies in natural processes, i.e., chromatin packaging and incomplete apoptosis [66] could be involved in DNA damage in germ cells.

Alkaline comet assay is a preferred method of choice when measuring low levels of primary DNA damage, as is the case in our study [67]. It can sensitively quantify DNA damage on a cell-by-cell basis, which gives this test an advantage over other cytogenetic techniques [68]. Our results indicated that the oral exposure of male rats to 0.06, 0.8, and 2.25 mg/kg b. w./day for 28 consecutive days caused a low level of DNA damage in testicular sperm cells with no obvious dose-response. The higher the percentage of DNA in the tail of comets found in imidacloprid treated groups suggests the formation of breaks in heritable DNA. Considering that no significant changes in oxidative stress response were observed in testicular tissue, we can presume that direct toxic effects were responsible for the genome instability as measured by the alkaline comet assay. Imidacloprid reacting with nucleic acid forms an ion-association complex, while the pyridine group of imidacloprid can interact with the nucleic acid base [69]. Such interactions may lead to an instability of the DNA molecular structure and contribute to the infliction of breaks. The lack of a dose-response relationship can indicate that the observed significant increase of comet tail intensity is not caused by imidacloprid. However, the absence of a dose-response relationship may also be explained by the fact that, although actual DNA damage is possibly greater at higher imidacloprid doses, these highly damaged cells are lost from scoring. Namely, we excluded nucleoids whose tail length was greater than 80 and head intensity was lower than 60, and it is possible that the remaining cells measurable by the image analysis system had less DNA damage. Such results could be the consequence of the highly fragmented nucleoids originating from apoptotic cells that could be “washed out” from agarose gel during electrophoresis. Therefore, the obtained values based on less damaged nucleotides would be lower than real damage. Such an influence of apoptosis on DNA damage was established in earlier comet assay studies [70,71]. In addition, the slower double-strand breaks repair half-life in low-dose exposure than in that of higher/high dose exposure [72] may contribute to the absence of dose-response relationship.

Differences in DNA damage levels measured in rat testicular and epididymal cells might be associated with intrinsic differences between these cell types and the efficiency of their DNA repair mechanisms. The results obtained for the epididymis are in agreement with the ones obtained for GSH and antioxidant enzymes, suggesting that oxidative stress contributes to the infliction of DNA damage, which has also been reported in several previous studies that evaluated the genotoxic potency of imidacloprid at much higher doses [27,31,60]. Taken together, the results suggest that the exposure to low doses of imidacloprid may pose a certain risk at the genome level, and with the induction of oxidative stress responses, it contributes to DNA instability.

**Figure 4:**
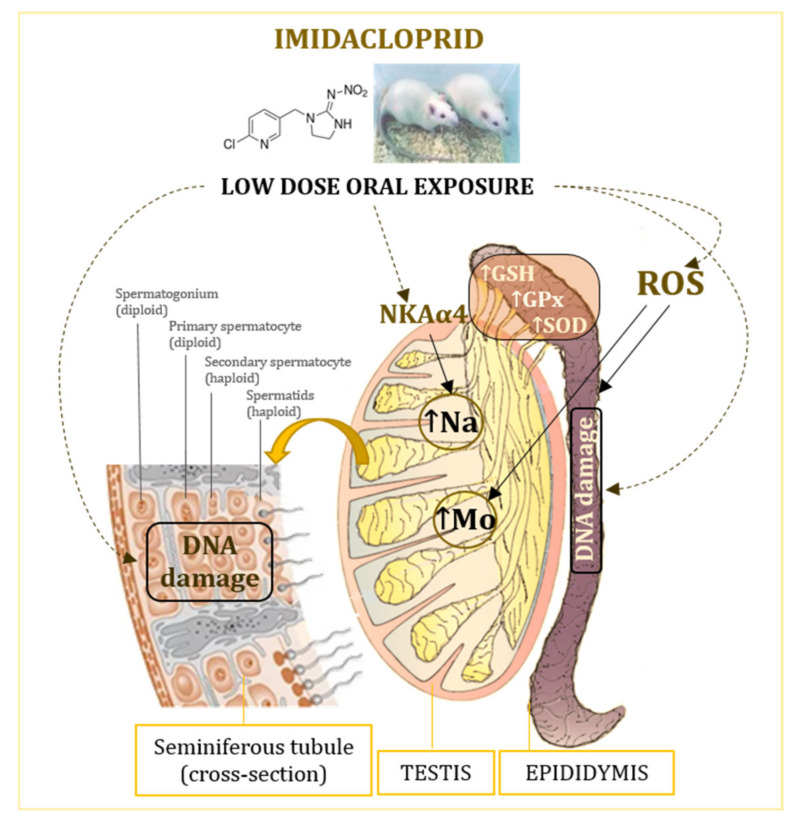
Effects of oral exposure to low doses of imidacloprid on the testis and epididymis of adult male Wistar rats. GSH—glutathione; GPx—glutathione peroxidase; NKAα4—isoform of the Na, K-ATPase catalytic subunit; ROS—reactive oxygen species; SOD—superoxide dismutase.

The proposed mechanisms of low-level imidacloprid adverse effects in the testes and epididymis are shown in Figure 4.

## 5. Conclusions

It is important to emphasize that, in comparison to previous research, these results were obtained at very low imidacloprid doses relevant for the real scenario exposure assessed by regulatory agencies. Measurable biological effects, although not clearly related to increased imidacloprid dose, indicate the potential hazards posed by this widely used insecticide and highlights the importance of protective measures and safety regulations to minimize exposure. Although the member states of the European Union decided to accept the recommendation proposed by the European Commission and restricts its use within open field agricultural industry, imidacloprid can still be used in enclosed greenhouses as well as in many veterinary drugs [73]. As residues of imidacloprid can be found in frequently consumed food and water, and human exposure usually involves a mixture of various chemicals that can act in an additive or even synergistic manner, the impact of a low dose of imidacloprid and other pesticides on male reproductive health warrants further investigation. Additional studies to investigate the role of oxidative stress in toxic effects of low-level imidacloprid exposure are required.

## Figures and Tables

**Table 1:** Concentration of essential elements in the testis and epididymis of Wistar rats (N = 5 rats per group) treated orally for 28 consecutive days with imidacloprid at doses of 0.06 mg/kg b. w./day, 0.8 mg/kg b. w./day, and 2.25 mg/kg b. w./day and the respective negative control ^1^.

		Negative Control	0.06 mg/kg b. w./day	0.8 mg/kg b. w./day	2.25 mg/kg b. w./day
TESTIS					
Na	mg/kg	1555 (1503–1653)	1541 (1516–1588)	1646 (1540–1715)	1695 (1545–1767) ^a,b^
Mg		198 (171–213)	195 (176–212)	201 (179–209)	201 (168–225)
Ca		52.3 (49.3–74.8)	50.3 (44.2–56.7)	51.9 (48.7–60.5)	54.0 (49.7–85.2)
K		4282 (3805–4420)	4272 (3798–4597)	4378 (3819–4447)	4311 (3661–4557)
Fe		24.2 (21.1–26.8)	24.9 (22.9–26.8)	25.0 (24.6–26.2)	24.4 (21.5–30.6)
Cu		2.14 (1.85–2.25)	2.01 (1.88–2.23)	2.04 (2.00–2.11)	2.08 (1.67–2.30)
Zn		27.7 (25.9–28.5)	27.3 (24.9–29.8)	27.8 (25.1–28.9)	27.9 (18.6–32.1)
Se	μg/kg	966 (940–996)	925 (859–1021)	965 (852–997)	965 (671–1162)
Mo		60.6 (51.3–72.4)	59.0 (55.8–65.0)	76.5 (58.8–76.9) ^a,b^	72.5 (63.2–88.2) ^a,b^
Mn		331 (295–376)	321 (300–381)	327 (317–341)	337 (272–477)
EPIDIDYMIS					
Na	mg/kg	1038 (985–1292)	1416 (971–1472)	1331 (1210–2182)	1338 (1209–1463)
Mg		110 (76.3–128)	129 (83.7–174)	128 (90.8–234)	114 (98.3–135)
Ca		72.7 (51.4–99.2)	65.4 (61.6–72.6)	79.8 (64.5–90.5)	66.5 (60.1–102)
K		1469 (1172–1794)	1875 (1237–2154)	1724 (1396–3291)	1620 (1544–1911)
Fe		18.2 (17.6–21.0)	19.1 (16.9–21.0)	18.3 (16.3–29.8)	19.9 (17.1–28.9)
Cu		1.37 (1.16–1.85)	1.70 (1.08–1.91)	1.46 (1.32–2.95)	1.58 (1.09–1.61)
Zn		24.1 (22.4–32.3)	30.5 (17.8–36.5)	27.0 (18.6–55.4)	27.3 (24.1–28.8)
Se	μg/kg	851 (791–1131)	1003 (712–1255)	916 (652–1877)	968 (828–1015)
Mo		56.7 (53.1–67.5)	72.7 (51.4–82.4)	71.8 (58.0–76.8)	68.6 (65.2–82.3)
Mn		203 (178–258)	260 (164–286)	237 (172–441)	219 (207–298)

^1^ Results are presented as median and range (min–max). Statistical significance was set at *p* < 0.05 (a—vs. negative control, b—vs. dose 0.06 mg/kg b. w./day).

## Data Availability

Data is contained within the article and Supplementary Material.

## Supplementary Materials

The following are available online at https://www.mdpi.com/article/10.3390/antiox10121965/s1, Table S1: Weight of testis/epididymis, antioxidant parameters and concentration of essential elements in tissues (testis, epididymis) and % of DNA damage in testicular haploid/diploid and epididymal sperm cells of Wistar rats (N = 5 rats per group).
