# Supplementary material for: Effects of Sub-Chronic Exposure to Imidacloprid on Reproductive Organs of Adult Male Rats: Antioxidant State, DNA Damage, and Levels of Essential Elements
Source: Antioxidants (Basel). 2021 Dec 8;10(12):1965. doi: 10.3390/antiox10121965 (PMC8750738; doi:10.3390/antiox10121965)
Supplement: Supplementary file 1 [file antioxidants-10-01965-s001.zip › antioxidants-1478980-supplementary.pdf]

Table S1. Weight of testis/epididymis, antioxidant parameters and concentration of essential elements in tissues (testis, epididymis) and % of DNA damage in testicular haploid/diploid and epididymal sperm cells of Wistar rats (N=5 rats per group). Rats are treated orally for 28 consecutive days with imidacloprid at doses of 0.06 mg/kg b. w./day, 0.8 mg/kg b. w./day and 2.25 mg/kg b. w./day. Data are expressed as mean ± standard deviation, median and range (min – max).

	NEGATIVE CONTROL	0.06 mg/kg b. w./day	0.8 mg/kg b. w./day	2.25 mg/kg b. w./day
		ORGAN WEIGHT		
**TESTIS (g)**	1.62 ± 0.15	1.56 ± 0.13	1.48 ± 0.13	1.30 ± 0.17
	1.56 (1.48-1.80)	1.62 (1.38-1.68)	1.48 (1.38-1.57)	1.27 (1.08-1.49)
**EPIDIDYMIS (g)**	0.865 ± 0.106	0.809 ± 0.061	0.741 ± 0.084	0.792 ± 0.090
	0.807 (0.778-1.019)	0.790 (0.744-0.891)	0.729 (0.624-0.858)	0.774 (0.708-0.918)
		ANTIOXIDANT PARAMETERS		
**TESTIS**				
**GSH (μg/g tissue)**	12.3 ± 1.3	11.3 ± 1.4	12.3 ± 1.5	11.1 ± 3.1
	12.4 (10.6-13.7)	10.8 (9.86-13.5)	12.1 (10.8-14.5)	11.1 (7.38-14.7)
**GPx (U/g protein)**	179 ± 55	219 ± 65	279 ± 72	206 ± 67
	179 (127-231)	216 (158-319)	307 (197-333)	193 (135-308)
**SOD (U/g protein)**	12269 ± 3222	15940 ± 870	14111 ± 1784	13072 ± 1355
	11726 (9352-15727)	15659 (15244-16916)	14463 (12177-15692)	12865 (11832-14519)
**EPIDIDYMIS**				
**GSH (μg/g tissue)**	1.33 ± 0.19	2.30 ± 0.60	1.68 ± 0.60	1.35 ± 0.36
	1.41 (1.06-1.50)	2.19 (1.61-3.23)	1.71 (0.95-2.49)	1.29 (0.84-1.70)
**GPx (U/g protein)**	281 ± 55	487 ± 70	432 ± 79	539 ± 57
	269 (232-351)	483 (415-568)	429 (335-513)	538 (471-598)
**SOD (U/g protein)**	17501 ± 1036	24347 ± 2534	22532 ± 2505	22008 ± 1387
	17057 (16315-18858)	24079 (22283-28626)	22017 (20525-26826)	22561 (20031-23511)
		ESSENTIAL ELEMENTS		
**TESTIS**				
**Na (mg/g)**	1562 ± 56	1546 ± 27	1636 ± 67	1679 ± 82
	1555 (1503 – 1653)	1541 (1516 – 1588)	1646 (1540 – 1715)	1695 (1545 – 1767)
**Mg (mg/g)**	194 ± 16	194 ± 16	197 ± 11	200 ± 21
	198 (171 – 213)	195 (176 – 212)	201 (179 – 209)	201 (168 – 225)
**Ca (mg/g)**	56.1 ± 10.5	49.8 ± 4.7	53.0 ± 4.5	59.8 ± 14.5
	52.3 (49.3 – 74.8)	50.3 (44.2 – 56.7)	51.9 (48.7 – 60.5)	54.0 (49.7 – 85.2)
**K (mg/g)**	4149 ± 302	4199 ± 362	4280 ± 260	4156 ± 361
	4282 (3805 – 4420)	4272 (3798 – 4597)	4378 (3819 – 4447)	4311 (3661 – 4557)
**Fe (mg/g)**	24.3 ± 2.1	24.6 ± 1.6	25.3 ± 0.7	25.0 ± 3.4
	24.2 (21.1 – 26.8)	24.9 (22.9 – 26.8)	25.0 (24.6 – 26.2)	24.4 (21.5 – 30.6)
**Cu (mg/g)**	2.09 ± 0.16	2.04 ± 0.13	2.05 ± 0.04	2.05 ± 0.23
	2.14 (1.85 – 2.25)	2.01 (1.88 – 2.23)	2.04 (2.00 – 2.11)	2.08 (1.67 – 2.30)

**Zn (mg/g)**	27.3 ± 1.0	27.3 ± 2.1	27.4 ± 1.5	26.3 ± 5.2
	27.7 (25.9 – 28.5)	27.3 (24.9 – 29.8)	27.8 (25.1 – 28.9)	27.9 (18.6 – 32.1)
**Se (µg/g)**	967 ± 21	937 ± 68	949 ± 58	925 ± 184
	966 (940 – 996)	925 (859 – 1021)	965 (852 – 997)	965 (671 – 1162)
**Mo (µg/g)**	60.5 ± 7.8	59.6 ± 3.5	72.9 ± 7.9	73.9 ± 9.1
	60.6 (51.3 – 72.4)	59.0 (55.8 – 65.0)	76.5 (58.8 – 76.9)	72.5 (63.2 – 88.2)
**Mn (µg/g)**	332 ± 34	326 ± 33	329 ± 10	359 ± 76
	331 (295 – 376)	321 (300 – 381)	327 (317 – 341)	337 (272 – 477)
**EPIDIDYMIS**				
**Na (mg/g)**	1127 ± 148	1319 ± 236	1477 ± 398	1314 ± 107
	1038 (985 – 1292)	1416 (971 – 1472)	1331 (1210 – 2182)	1338 (1209 – 1463)
**Mg (mg/g)**	108 ± 21	129 ± 37	141 ± 55	116 ± 13
	110 (76.3 – 128)	129 (83.7 – 174)	128 (90.8 – 234)	114 (98.3 – 135)
**Ca (mg/g)**	71.5 ± 17.9	66.2 ± 5.0	77.6 ± 12.3	77.4 ± 19.2
	72.7 (51.4 – 99.2)	65.4 (61.6 – 72.6)	79.8 (64.5 – 90.5)	66.5 (60.1 – 102)
**K (mg/g)**	1499 ± 247	1785 ± 390	2009 ± 741	1669 ± 145
	1469 (1172 – 1794)	1875 (1237 – 2154)	1724 (1396 – 3291)	1620 (1544 – 1911)
**Fe (mg/g)**	19.1 ± 1.7	19.0 ± 1.8	21.0 ± 5.4	21.0 ± 4.7
	18.2 (17.6 – 21.0)	19.1 (16.9 – 21.0)	18.3 (16.3 – 29.8)	19.9 (17.1 – 28.9)
**Cu (mg/g)**	1.45 ± 0.26	1.59 ± 0.37	1.78 ± 0.67	1.48 ± 0.22
	1.37 (1.16 – 1.85)	1.70 (1.08 – 1.91)	1.46 (1.32 – 2.95)	1.58 (1.09 – 1.61)
**Zn (mg/g)**	26.5 ± 4.8	28.9 ± 8.4	31.5 ± 14.0	26.9 ± 2.1
	24.1 (22.4 – 32.3)	30.5 (17.8 – 36.5)	27.0 (18.6 – 55.4)	27.3 (24.1 – 28.8)
**Se (µg/g)**	937 ± 169	993 ± 238	1062 ± 475	945 ± 81
	851 (791 – 1131)	1003 (712 – 1255)	916 (652 – 1877)	968 (828 – 1015)
**Mo (µg/g)**	58.8 ± 5.4	69.8 ± 13.3	69.6 ± 8.3	71.0 ± 6.6
	56.7 (53.1 – 67.5)	72.7 (51.4 – 82.4)	71.8 (58.0 – 76.8)	68.6 (65.2 – 82.3)
**Mn (µg/g)**	208 ± 32	242 ± 57	276 ± 103	238 ± 39
	203 (178 – 258)	260 (164 – 286)	237 (172 – 441)	219 (207 – 298)
		**% DNA DAMAGE**		
**HAPLOID TESTICULAR CELLS**	1.81 ± 2.73	2.53 ± 3.16	2.60 ± 3.16	2.32 ± 3.01
	0.421 (0 - 13.9)	1.14 (0 - 16.0)	1.25 (0 - 18.4)	1.06 (0 - 16.8)
**DIPLOID TESTICULAR CELLS**	1.68 ± 2.24	2.37 ± 2.87	2.26 ± 2.66	2.32 ± 2.96
	0.587 (0 - 15.4)	1.16 (0 - 18.8)	1.15 (0 - 14.1)	1.00 (0 - 15.7)
**EPIDIDYMAL CELLS**	2.36 ± 2.93	3.15 ± 3.34	2.38 ± 3.00	2.35 ± 2.98
	1.06 (0 - 18.0)	2.02 (0 - 16.0)	0.977 (0 - 14.9)	1.11 (0 - 17.3)
